# Unpacking the root causes of gambling in the Asian community: Contesting the myth of the Asian gambling culture

**DOI:** 10.3389/fpubh.2022.956956

**Published:** 2022-11-03

**Authors:** Mia Han Colby, Ben Hires, Lisette Le, Dawn Sauma, Man Yoyo Yau, MyDzung Thi Chu, Heang Leung Rubin

**Affiliations:** ^1^Asian Center for Addressing Research, Education, and Services (CARES), Boston, MA, United States; ^2^ADAPT Coalition, Clinical and Translational Science Institute, Tufts University, Boston, MA, United States; ^3^Boston Chinatown Neighborhood Center (BCNC), Boston, MA, United States; ^4^Vietnamese American Initiative for Development (VietAID), Dorchester, MA, United States; ^5^Asian Task Force Against Domestic Violence (ATASK), Boston, MA, United States; ^6^Tufts Medical Center, Institute for Clinical Research and Health Policy Studies, Boston, MA, United States

**Keywords:** gambling, anti-Asian racism, community-based organizations, culturally and linguistically appropriate services, health equity, community fieldworkers, community-based participatory research (CBPR), integration

## Abstract

**Introduction:**

Problem gambling is a public health issue both in the United States and internationally and can lead to mental health and socioeconomic concerns for individuals, families, and communities. Large epidemiological studies on problem gambling have neglected to include working-class, immigrant Asian Americans, who are at higher risk for problem gambling. The lack of data on Asian American gambling may explain a subsequent lack of culturally and linguistically appropriate treatment and prevention services. Additionally, the invisibility of Asian American data in published literature has helped to perpetuate a commonly held myth of an Asian gambling culture. This stereotype of the “Asian gambler” is a form of anti-Asian racism which serves to ignore and minimize the root causes of problem gambling in the Asian American community.

**Methods:**

Utilizing a community-based participatory research approach, 40 interviews were conducted with the local Khmer (*n* = 12), Chinese (*n* = 20), Korean (*n* = 3), and Vietnamese (*n* = 5) immigrant communities in the Greater Boston region to assess how problem gambling manifests in the local Asian community. Interviews were conducted in language by bilingual/bicultural community fieldworkers experienced in serving their respective communities. Flyers and social media were used to recruit participants. The interviews were coded into themes which provided a better understanding of the patterns of systemic issues contributing to problem gambling in the Asian American community.

**Results:**

Interviewees provided insights into the underlying issues of poverty and social and cultural loss due to immigration as root causes for problem gambling in the Asian American community. The interviews indicate that many individuals in these Asian immigrant communities, who are striving to make a living off low-wage and stressful jobs, struggle to integrate into American society. They often lack culturally appropriate and accessible social and recreational activities, a void that casinos capitalize on through targeted behaviors.

**Discussion:**

Research must address the social and structural barriers in the Asian American communities rather than relying on the “Asian gambler” stereotype and assuming interventions for a general American problem gambler will work for Asian immigrants. The research points to a need for gambling interventions and services that are centered on lived experiences.

## Introduction

Problem gambling is a pervasive social, economic, and public health issue in the Asian immigrant working-class communities. Amongst the general population, problem gambling is associated with financial harms, relationship disruption, family and intimate partner violence, and emotional and psychological distress (1–3). Beyond these potential harms, current research indicates social and economic impacts of problem gambling on Asian American communities and families (3–7). A growing body of literature suggests that the Asian community is at greater risk for problem gambling than the general public (8–10). In a US national survey conducted from 2001 to 2002, data showed that while only 4.4% of the sample population were Asian, 7.2% of the pathological gamblers surveyed were Asian, indicating that Asians were at higher risk for gambling disorders (11).

Despite the seriousness of problem gambling in Asian communities, large epidemiological studies on problem gambling in the US often neglect these communities, failing to capture the realities Asian immigrants are facing. Specifically for Massachusetts, a statewide study on gambling was unable to capture the Asian American demographic due to having too small a sample size (12). Most large studies are conducted in English, which fails to reach limited-English speaking and often lower-income Asians at higher risk for problem gambling. The lack of data inhibits the allocation of culturally and linguistically appropriate treatment and prevention services to address problem gambling in the Asian communities in the US. The invisibility of Asians in the data is a form of structural racism that leads to underserved communities and incomplete explanations of the issues that Asian communities face in the US.

In the absence of definitive research, the common myth of an “Asian gambling culture” has often been used as the driving explanation for Asian gambling which overshadows systemic and structural root causes of problem gambling in the Asian community. The popular stereotype is pervasive in the media and even a misconception found within the Asian community itself. For example, in 2011, the New York Times released an article which stated, “culture is one reason gambling is so popular among Asian-Americans […] Asian-Americans, carrying on a tradition from their homelands, embrace games of chance and skill like mah-jongg […] Las Vegas has long counted on a strong Asian clientele” (13). Cultural conceptions of luck or fate are often brought up as social norms that influence Asian gamblers (14–16). While these are societal beliefs in many Asian cultures, focusing solely on these factors overshadows other important root causes of problem gambling in the Asian community. The media especially has highlighted or sensationalized these cultural elements often titling articles or using the first few sentences to drive home the idea that Asians Americans gambling addiction is rooted in culture (17). Additionally, films, popular culture, and even casinos perpetuate the “Asian gambling stereotype” through their portrayals of Asian gamblers (18, 19). While academic research has made strides toward critiquing and countering this myth (20–22), the pervasiveness of the Asian gambler continues to infiltrate research and even casual conversation around the topic. The Asian gambler stereotype simplifies a complex issue and neglects to consider systemic problems related to poverty, immigration, and cultural integration. By focusing attention on the Asian gambler stereotype, this complex issue cannot be fully understood and we fail to properly protect Asian communities from gambling related harms. Complicating these issues, gambling addiction and problem gambling are especially stigmatized in Asian communities where the concept of face is particularly important (21, 23–25).

Community-based participatory research (CBPR) and community-engaged research (CER) have tried to address these data gaps by actively working with community-based organizations (CBOs) to research and investigate important issues in the community. CER is a process that involves the community as partners in research including the development of research questions, collection of data and subsequent analysis, as well as the dissemination of the research (26, 27). Through their involvement, these community partners provide expertise on their community and bring their own lived experience. CER has grown in recognition as a process that can help scientific discoveries lead to improved outcomes, especially for populations historically underrepresented in public health research. By utilizing the CER process, the knowledge and experience of the community aids in ensuring research priorities are responsive to the needs of the community and allows for the development of culturally sensitive practices and measures (28–30). Utilizing CBPR and CER focuses on the value and importance of CBOs in research.

Asian CARES (Center for Addressing Research, Education, and Services) is a coalition of ethnic specific CBOs in the Greater Boston area formed to address gaps in research and services addressing problem gambling and other public health challenges in the Asian^1^ communities. Over the years, and in the wake of the COVID-19 pandemic and rising anti-Asian racism (31–37), these agencies have seen an increasing trend of families seeking aid for financial problems stemming from problem gambling.

Asian CARES research was guided by CER and used these principles throughout the research process. The community-based partner organizations are multi-service agencies that offer a variety of services from childcare and English as a second language classes to workforce development and youth programming. These CBOs serve as trusted entities within their ethnic communities serving as a point of first contact between community members in need and mainstream organizations. As ethnic serving social service agencies, these partners help them navigate systems such as education, healthcare, and law.

Previous research on problem gambling in the Asian community of Boston's Chinatown was conducted by Dr. Carolyn Wong from the Institute of Asian American Studies at UMass Boston. The report emphasized the responses of 23 participants from Boston Chinatown who spoke about the protective and risk factors associated with problem gambling among the Chinese population (22). Importantly, Dr. Wong began the work of contesting the Asian gambler stereotype concerning the Chinese community, centering the issue of problem gambling within a deeper context than the racist focus on Chinese culture.

The research of this study builds upon Dr. Wong's findings attempting to further untangle the issue of problem gambling, specifically focused on expanding the research to other Asian ethnicities and delving deeper into problem gambling in the Asian community. Particularly, the objective of this study is to focus on community perceptions of gambling within each ethnic community rather than specific experiences of individuals with gambling problems.

## Materials and methods

### Community-engaged research coalition

The Asian CARES coalition served as the research team and advisory board for the study. These members were composed of multi-disciplinary partners including four CBO directors from our partner organizations, three researchers from Tufts University, and eighteen trusted community bilingual/bicultural fieldworkers. Each member of the research team played a critical role in the research. The researchers had expertise in CER in the Asian community. The community partners included Boston Chinatown Neighborhood Center, Asian Task Force Against Domestic Violence, Cambodian Mutual Assistance Association of Greater Lowell, and the Vietnamese American Initiative for Development.

The coalition met monthly, starting in late 2019, to guide the research and discuss findings. During these meetings, they contributed to the conception and implementation of the research design, provided important feedback about relevancy and wording of research questions to ensure that they were asked in a culturally-relevant way and tailored to the ethnic populations. Coalition meetings allowed researchers to gain a deeper understanding of each community and the issues they were dealing with. Considering the COVID-19 pandemic and subsequent rising racial tension, the meetings also served as a place for community partners to share what they were hearing from their constituents. Beyond serving as an advisory board for the research, the coalition also strove to amplify the issue of problem gambling in the Asian community.

### Instruments

The semi-structured interview protocol was developed using qualitative methods to determine “how” and “why” questions related to problem gambling (38–41). The interview guide (see Supplementary material) was broken into four sections which included questions on (1) Perceptions of gambling (2) Treatment options and solutions in the community (3) Coalition organizations (4) Demographics. The questions around perceptions of gambling ranged from more general questions about which types of gambling the participants felt was more prevalent to questions on their thoughts on gambling, gambling in their ethnic communities, and the impacts of gambling on families and their communities. Questions around treatment and solutions were designed to understand both the participants' knowledge of treatment options and their opinions on what they felt would be helpful for their specific ethnic communities. While the questions were designed to obtain an understanding of gambling, no one was asked to name or identify anyone they know who gambles. The demographic questions were designed to invoke non-identifying data and some questions were made optional to ensure privacy. While participants were asked whether they were an immigrant and their length of time in the US, emphasis was placed on ensuring participants did not need to disclose their immigration status or whether they were legal immigrants.

All instruments, including community fieldworker training materials, recruitment materials, the interview protocol, and the consent script were approved by the Tufts University IRB. Instruments were translated into several Asian languages including Chinese, Vietnamese, Khmer, and Korean.

### Training of fieldworkers

Community fieldworkers, recruited from the bilingual/bicultural staff of the partner agencies, were trained to conduct the interviews with participants. These community fieldworkers have already built trusting relationships with community members and were therefore the ideal agents to conduct interviews with their community. In light of the stigma of problem gambling within the community, it was important to ensure participants felt comfortable with the interviewer. Other community engaged research has used community fieldworkers for similar reasons (42). Trainings and interview protocols were provided to the community fieldworkers to guide their discussions.

Two IRB-approved research trainings were developed and administered to the community fieldworkers. The first training covered human subjects and the ethical obligations of the community fieldworkers, as covered by the human subject's requirement of the Collaborative Institutional Training Initiative training. The second training covered interviewing techniques. Trainings took place remotely via Zoom.

Community fieldworkers were provided with materials to guide the interview process including a step-by-step interview process checklist, informed consent forms, informed consent documentation logs, and the interview question guidelines. The interview protocol was used to guide the interview process. Community fieldworkers were trained to adapt to the situation of the interview and probe participants to elaborate on their answers.

To ensure consistency of data collection across ethnic groups, a weekly meeting was held with all the community fieldworkers during the data collection phase. The supervision meetings allowed the researchers to monitor recruitment and troubleshoot any emerging problems during the interview process.

### Recruitment methods

Outreach was done through the Asian CARES partner organizations. The recruitment process resulted in a convenience sample as those recruited were from the ethnic groups served by the current coalition partners. Translation of recruitment documents into these four languages was due to Chinese, Vietnamese, Khmer, and Korean ethnicities representing some of the larger Asian immigrant communities in the Greater Boston area. While recruitment was attempted with other Asian ethnic groups, there was little to no participant response (see Table 1).

**Table 1:** Demographics information of the interview participants.

**Demographic variables**	**Number**	**Percent**
**Race/Ethnicity**	***N*** **=** **35**	
Chinese	15	43%
Chinese Vietnamese	1	3%
Filipino	1	3%
Khmer	10	29%
Korean	3	9%
Taiwanese	1	3%
Vietnamese	4	11%
**Gender**	***N*** **=** **33**	
Male	9	27%
Female	24	73%
**Education**	***N*** **=** **34**	
Grade school	2	6%
High school or equivalent	15	44%
Associates degree	1	3%
Bachelor's degree	11	32%
Master's degree or professional degree	5	15%
**Primary language**	***N*** **=** **34**	
Cantonese	5	15%
English	8	24%
Khmer	7	21%
Korean	2	6%
Mandarin	10	29%
Vietnamese	4	12%
Other (Tagalog)	1	3%
**English proficiency**	***N*** **=** **33**	
Fluent	8	24%
High	3	9%
Medium	8	24%
Limited	14	42%
None	0	0%
**Age**	***N*** **=** **39**	
Under 20	1	3%
20–29	7	18%
30–39	12	31%
40–49	10	26%
50–59	7	18%
60–69	1	3%
70+	1	3%
**Migrated**	***N*** **=** **37**	
Yes	34	92%
No	3	8%

Participants could choose to decline questions they were not comfortable answering. Some participants were bilingual and chose more than one language as their primary language. For analysis purposes, the Taiwanese identifying participant was included in the Chinese group and the Chinese Vietnamese identifying participant was included in the Vietnamese group.

Recruitment of interview participants mainly relied on distribution of a standard recruitment flyer both in physical distribution and through social media, but a recruitment script was also developed for more direct outreach through email or verbal contact. Response to recruitment was rapid for many agencies and required a vetting process. Recruitment focused on adult (over the age of eighteen) community members who had a family member, neighbor, or co-worker who gambled, and the participant was able to discuss their observations and experiences with gambling behavior. Gambling was not an inclusion or exclusion criteria for this study and many participants had some personal gambling experience. As such, no mechanisms were used to determine the problem gambling severity status of the participant. However, self-identifying problem gamblers were excluded from the study as the community fieldworkers did not have the qualifications to conduct research with such a high-risk group. Additionally, participants were screened for their geographic location and excluded if they were beyond the catchment area for the study. Focusing on community members and their experiences allowed a broader view of gambling in the Asian community as a whole and allowed a clearer understanding of perceptions of gambling within the community.

A standard, recruitment flyer was developed and then translated into several Asian languages including Chinese, Vietnamese, Khmer, and Korean.

The recruitment flyer was distributed by community partners and the community fieldworkers as part of their normal contact with clients through channels like program emails and social media posts. While some participants were recruited through direct contact distribution, due to the need for social distancing, participants were mainly recruited by indirect contact. Mainstream American social media platforms such as Facebook and Instagram were used; however, agencies also chose to use community group text channels and more Asian specific social media platforms such as WeChat. Social media is a common way for the community agencies to communicate with their clients and constituents which the community partners emphasized as an important tool to reach a broader range of stakeholders.

### Interview process

From February to April of 2021, the community fieldworkers conducted forty interviews of residents from the Khmer, Chinese, Korean, and Vietnamese immigrant communities in the Greater Boston area to assess how problem gambling manifests in their communities. These interviews were conducted in language by bilingual/bicultural community field workers experienced in serving their respective communities. Because of the increased strain the community fieldworkers were experiencing due to supporting the Asian community through the racial tensions due to the COVID-19 pandemic and the shootings in Atlanta (43–45), the number of interviews was limited to forty. Forty interviews were deemed a reasonable number to expect of the already overstretched workforce and forty within the bounds of adequate saturation for qualitative interviewing (46–49).

Semi-structured interviews took place through Zoom or the phone and lasted between 45 and 60 min. Participants received a $50 gift card for their participation. Interviews were audio and/or video recorded with permission. Some participants elected to not be recorded, in which case the community fieldworker took notes during the interview. Community fieldworkers utilized the interview protocol to guide their interview process. When possible, interviews were conducted in pairs with one fieldworker taking the lead to conduct the interview and another taking notes. After each interview recordings were reviewed by the community fieldworkers and notes were revised to verify accuracy. Interviews were conducted in the language the participant felt most comfortable with. The community fieldworkers provided English translations of the notes for the researchers to analyze.

To ensure uniformity and consistency of the data collected across interviews, the primary investigator, who has expertise in qualitative interviewing, reviewed the notes from each community fieldworker's first interview. Additionally, when reviewing notes, the primary investigator was able to evaluate the interview questions and adjust based on cultural understandings. For example, the concept of boredom became apparent in early interviews and the interview guide was adjusted to account for a need to probe around what “boredom” meant. The primary investigator also took time to ensure fieldworkers were probing interview participants where needed. After the notes were taken and approved, the audio and/or video recordings were destroyed to ensure the privacy of the interview participants.

### Data analysis interview coding

The interviews were coded and developed into themes that described patterns of systemic issues contributing to problem gambling in the Asian community. Data analysis drew upon elements of thematic analysis and the framework method (50–52). Coding and data analysis was performed by two researchers. Before analysis, a list of priori codes was developed based on research literature and used as the basis for a codebook. The researchers initially coded independently for two rounds before meeting to discuss their findings and determine main codes. The first round of coding was done using these prior deductive codes to determine consistencies between the research findings and the literature. A second round of coding focused on inductive codes and narratives in the interviews which were unique to the Asian community (53).

The codes were then developed into themes. Attention was paid to themes related to understanding the effects of gambling in the Asian community. The themes served as primary building blocks for understanding patterns of systemic issues contributing to gambling and the ways these systemic issues manifest in the Asian community (54–59). Preliminary analysis was presented to the community fieldworkers for their feedback and to ensure researcher interpretation was accurate to the data collected.

## Results

Interviewees provided insights into the underlying issues of poverty and social and cultural loss due to immigration as root causes for problem gambling in the Asian community. The interviews indicate that many individuals in these Asian immigrant communities were striving to make a living off low wage and stressful jobs and struggled to integrate into American society. They often lacked culturally appropriate and accessible social and recreational activities, a void that casinos capitalize on through targeted behaviors.

Table 1 details the demographic information of the forty interview participants. The majority of participants were immigrants (92%) who identified as having a medium English proficiency or less (66%). Of these participants, 50% had a high school diploma or less and 52% worked in the services industry. Interviews spanned five ethnic groups – Chinese (46%), Khmer (29%), Vietnamese (14%), Korean (9%), and Filipino (3%). All interviewees drew from direct experiences of family members, friends, co-workers, and neighbors who gamble.

Prominent themes (see Table 2) were found through the data analysis process involving systemic issues which contributed to gambling in the Asian community. These themes include cultural and linguistic barriers, poverty, stress, desperation, and the influence of casinos and advertising.

**Table 2:** Participants identified reasons why they thought Asians gamble.

**Reasons to gamble**	**Total participants**		**Participants responses by ethnicity**							
	**Count**	**Percent**	**Vietnamese**		**Khmer**		**Chinese**		**Korean**	
			**Count**	**Percent**	**Count**	**Percent**	**Count**	**Percent**	**Count**	**Percent**
Social aspects	26	65%	3	12%	8	31%	13	50%	2	8%
Stress relief	9	23%	0	0%	2	22%	7	78%	0	0%
Obsession/greed	7	18%	2	29%	3	43%	1	14%	1	14%
Boredom	16	40%	1	6%	5	31%	10	63%	0	0%
No entertainment options	10	25%	0	0%	0	0%	10	100%	0	0%
Recreation	5	13%	1	20%	3	60%	0	0%	1	20%
Earn money/win money	23	58%	3	13%	10	43%	8	35%	2	9%
Improve family finance	7	18%	1	14%	2	29%	4	57%	0	0%
Social isolation	11	28%	1	9%	2	18%	8	73%	0	0%
Escape reality	6	15%	1	17%	1	17%	3	50%	1	17%

Reasons for gambling were coded and developed into the following themes. Each theme was analyzed in terms of the total participant count and then broken down into each ethnicity associated with that theme.

### Poverty, stress, and desperation

Poverty emerged as an important theme underlying what drives many to gamble. The theme was particularly salient in relation to the ways in which Asian immigrant communities are struggling to make a living off low wage and stressful jobs. These low-wage jobs were described by participants as difficult jobs where they cannot find meaning in the work they do. One participant speculated that there was “nowhere else to go other than work, they are unable to find other ways to make their life meaningful.” Participants connected poverty and being unable to make a decent living working low-wage jobs with stress, worsening mental health, and turning to gambling as both a stress reliever and a hopeful solution to their financial problems.

#### Gambling to earn money

The concept of gambling to earn money was frequently brought up by participants, with 58% mentioning gambling to earn quick or easy money. Alternatively, 18% of participants mentioned improving family finances as a reason for gambling. These two motivations were distinctly separated by participants, but both stem from a feeling of desperation and a desire to supplement income. An interviewee stated that gambling represents a “hope that they can have freedom of money” and was rooted in a desire to escape poverty. Gambling represented a dream of a better future.

#### Gambling to relieve stress

Gambling to relieve stress was a theme brought up by 23% of participants, all of whom were Chinese and Khmer. The concept is particularly important as stress relief was linked to work pressure. Participants described heavy workloads and long work hours that immigrants face in the US. Gambling became an outlet for stress. One Chinese respondent described the bleak situation of some immigrant restaurant workers who “work 12 h usually, come back home very late at night, such as at 11 p.m., and then they want to relax. It is year after year, day after day.” The participant went on to explain that casinos were one of the only relaxing activities available to restaurant workers who cannot find other recreational opportunities that are culturally and linguistically appropriate.

#### Depression

When discussing the stressors related to low-wage work, integration, and the challenges to make a living wage as an immigrant, participants started to talk about the connection between depression and gambling. Participants described the cycle of gambling where one becomes caught continually losing and accruing debt which leads to stress, depression, and desperation. In this state of desperation, more gambling can appear as the only viable way out. One participant spoke of depression and stated “Depression, when people are poor, they tend to have no way of getting out of it. They try to find the easiest way, which is gambling.” Others described the connection between worsening mental health as a gambler continues to gamble and lose money. A participant summed up the specific struggle of immigrants, “you have to borrow money, you can't pay the rent, you feel stressed, and you feel even more depressed.”

### Challenges to integrate into US society

The issue of cultural and linguistic barriers was apparent in several subthemes during the interviews. Particularly, language serves as a large barrier for integration and potential enjoyment of American recreational pastimes (movies, bars, theaters, sporting events, concerts, etc). One participant described a reluctance to go to a bar to relieve stress after work as the experience was not something they found relaxing and enjoyable, stating: “it is not easy to go to a bar to have a drink and find ways to entertain when they cannot speak the language.” Language barriers mean that something as simple as going to a bar or attending a movie are not accessible for limited English-speaking Asians.

Beyond simply linguistic barriers, the systemic issues related to culture and language and their relation to root causes of problem gambling resulted in the following themes:

#### Social isolation

The theme of social isolation was related to the issue of difficulty for immigrants to integrate into American society. Cultural and linguistic barriers serve as an isolating factor and 28% of participants indicated social isolation as a reason for gambling. Most of the interviewees (92%) were immigrants, for whom English was not their first language. Loneliness was a common word used by participants to describe reasons for gambling. One interviewee said gamblers “may feel lonely and [it is] hard to work in a foreign country” while another stated, gamblers “are alone, and unable to integrate into American society.” The same participant noted that “even [if] you don't speak English you can gamble.”

Additionally, 65% of participants mentioned the social aspect of gambling. Many immigrants leave their families behind in their countries of origin. One interviewee said that “People who don't have a family here will gamble […] when gambling there are many people chatting, contacting them.” Participants claimed that they would go to the casino as an activity with friends and that many people from the Asian community go to the casino.

#### Boredom

Though the concept boredom is related to social isolation, the continual use of the phrase prompted a deeper investigation. “Boredom” was a phrase used by 40% of participants and was particularly expressed in the Chinese and Khmer ethnic communities. When community fieldworkers probed participants to understand what boredom means, it became apparent that boredom was linked to the challenges with assimilating into American society. While on the surface, the theme of boredom could superficially be viewed as feeling like there is nothing else to do, the concept is strongly linked with the theme of a lack of culturally appropriate social outlets. Boredom stems from the challenge of integration and what one participant described as being “part of the life cycle of being an immigrant.” When asked to describe what they meant by the word boredom, one participant mentioned that the root of boredom was really the challenge of integrating into American culture. One participant wondered if “maybe gambling is a comfort” to immigrants who are feeling lonely in a foreign country. Another participant described the loneliness of being an immigrant: “Maybe it's because living here, they're so far from their homeland […] But maybe it's also living here, feeling so isolated and far from home…” Boredom reflects the loneliness and longing of a population who has left behind what they know and are struggling to adapt to foreign surroundings.

#### Lack of culturally appropriate social spaces

When discussing boredom, 25% of participants began to discuss the lack of social spaces in which they feel comfortable, causing community members to turn to gambling as an alternative. It should be noted that all the participants who mentioned lack of social spaces were Chinese respondents. The Chinese participants explained that in their native countries there are many entertainment options such as singing, karaoke, concerts, clubs, and other recreational opportunities (e.g., ping-pong or dancing). As immigrants in the US, they find it difficult to find activities that they are familiar with or activities that are in their language.

### Relation of gambling to casinos

Participants largely associated gambling with casinos when talking about gambling and gambling in their respective ethnic communities. Most respondents (78%) listed casino games as a type of gambling. When asked where gambling occurred, 83% of interviewees stated in casinos. The connection that participants felt between the casino and gambling is related to themes around activities promulgated by the casino.

#### Use of casino buses

Over 80% of interviewees were aware of buses in their communities which would bring individuals to the casino (see Table 3). One Khmer participant described the buses as “giv[ing] people the illusion that they are VIPS” providing great service and transporting people to a location where others spoke their language. In the Greater Boston area, casino buses are commonly stationed at known busy locations in local Asian ethnic enclaves such as Boston's Chinatown, Quincy, Dorchester, and Malden. Some casino buses to Boston's Chinatown run as frequently as every half hour and throughout the night.

**Table 3:** Participants were asked about their thoughts and experiences on gambling in their communities and potential triggers or facilitators to gambling.

**Triggers/ facilitators for gambling**	**Total participants**		**Participants responses by ethnicity**							
	**Count**	**Percent**	**Vietnamese**		**Khmer**		**Chinese**		**Korean**	
			**Count**	**Percent**	**Count**	**Percent**	**Count**	**Percent**	**Count**	**Percent**
Targeted advertising	17	43%	3	18%	2	12%	10	59%	2	12%
Casino environment	8	20%	1	13%	1	13%	5	63%	1	13%
Busing practices	34	85%	4	12%	10	29%	18	53%	2	6%
Entertainment turns to addiction	20	50%	2	10%	9	45%	7	35%	2	10%

The following themes were developed based on their responses. Each theme was analyzed in terms of the total participant count and then broken down into each ethnicity associated with that theme.

Some participants expressed what they felt was the strategic nature of the bus schedules. For example, one participant spoke of the buses being available once restaurant workers got off work. Considering the dearth of available recreation activities available to off work restaurant workers, the casino buses offered an easy and low-cost entertainment option for them to relieve stress. One participant spoke of friends in the restaurant industry who would get off a long stressful day of work, hop onto a bus to the casino, and then return on the bus before their next shift living a cycle of work to the casino and back.

#### Asian friendly casino environment

Many participants spoke to the environment within the casinos catering to an Asian clientele which made them feel welcome and comfortable. Two themes associated with the Asian friendly environment which were mentioned across all ethnicities were targeted and seductive advertising toward the Asian community (43%) and the ways in which the environment of the casino was designed to be particularly appealing to Asians (20%). Participants mentioned using free food and discounts to entice them, coupons were usually associated with taking the casino bus. The available food caters to Asian ethnicities and tastes. Several participants mentioned concerts, with one participant saying “the casino invites stars or singers from Hong Kong and Taiwan to sing” which draws in crowds. Beyond special events and activities, casinos provide a welcoming and Asian friendly environment. Participants mentioned croupiers and other casino employees who speak Asian languages (i.e., – Vietnamese, Mandarin, or Cantonese) providing a welcoming environment that immigrants may not find elsewhere. One interviewee mentioned that the “drivers and waiters who [work at] the casino make people feel comfortable and make you feel close to them.”

Participants described the role casinos played in encouraging Asians to come, gamble, and keep gambling. Participants from every ethnic group describe instances of free food vouchers which drew friends and family into casinos. Participants spoke to the temptation of the entertainment offered and the advertisements and offers which allow for free food and discount coupons which were described as “seduction.” From the perspective of the participants, they are being called or compelled to gamble rather than actively seeking the activity.

## Discussion

### Integration stress

The interviews illuminate systemic issues related to gambling in the Asian community, indicating a much more complex picture than the stereotypical view of Asians gambling culture. Delving deeper into the issue of gambling, root causes for problem gambling stemming from poverty to social and cultural isolation due to immigration became apparent.

The major themes related to reasons for problem gambling are largely intertwined with the challenges of integration and stress of immigrant life within American society. These stressors have left a toll on Asian immigrants and have been identified by participants as causes for problem gambling. Gambling is often expressed as a source of comfort or something people turn to as a replacement for something they feel they are lacking. The stress from work and daily life as an immigrant is exacerbated by a lack of outlets to relieve that stress.

Many working-class immigrants are struggling to learn English and find good paying jobs. Some experience downward mobility as they are unable to obtain equivalent jobs to what they had in their native countries. Community members have described work environments where working-class immigrants are working low-wage and stressful jobs which offer little hope of career advancement. In contrast, gambling offers what one participant described as a “false sense of accomplishment” and a “sense of fulfillment” which they are unable to find in their jobs. With a lack of opportunities and outlets to relieve stress and provide happiness, casinos and gambling were seen as a viable solution. Additionally, the stress, depression, and desperation associated with struggling to make a living led to a vicious cycle of gambling and worsening financial distress.

The concept of boredom and its links to social and cultural isolation are a perfect representation of how the Asian gambler is much more complex than it may look on the surface. The true meaning behind the word “boredom” indicates a complex and nuanced issue around the challenge to integrate into American culture and the lack of culturally appropriate and accessible recreational activities. Participants spoke of being unable to find the same kind of entertainment options they were used to in Asia and struggling to find places they felt comfortable. Boredom also implies that gambling is not the preferred activity, but rather the only activity they feel is available to them. Instead of saying Asians have a cultural propensity for gambling, it is more accurate to state that gambling fills a void for Asian immigrants seeking a refuge from their social and cultural isolation.

The previously described experience of a participant's reluctance to go to a bar to relax, is a great representation of the challenges of integration and the deeper complexity of Asian gambling. To an English speaker, going to a bar to relax and meet up with friends does not come with the stress and complexities that it does for an Asian immigrant with limited English capacity. Just ordering a drink or looking at a menu presents challenges and adds to stress. Rather than providing a relaxing environment, the barriers serve to emphasize otherness. The bar example also highlights further challenges for Asian immigrants participating in conventional American pastimes. For Asian immigrants working in the service industry, such as restaurant workers, most of the Asian language establishments (e.g., restaurants or bars) are closed by the time their shifts are over. Additionally, for an immigrant working a low-wage job and struggling to make a living, going to a bar may not be something they can afford with their salary. Many study participants highlighted the way gambling at casinos become an easy recreational activity due to the low threshold for entry. Participants noted that they did not need to know English to play slots, the drinks were free, and one only needed a little bit of money to start playing. Others noted that croupiers and other employees spoke Asian languages like Mandarin, Cantonese, or Vietnamese eliminating language barriers. Essentially, casinos have become a venue for Asian immigrants to relax, despite potential harms that gambling poses to Asians as a higher risk group.

Language barriers go beyond access to merely recreational activities. Rather these barriers create great challenges in many aspects of an immigrant's daily life including their ability to access services and navigate systems. The void that these immigrants feel reflects not only their struggle to integrate into American culture, but more importantly the failure of our society to accept Asian immigrants and their cultures. Rather the engagement that American society provides these vulnerable populations is in many ways directly harmful, going beyond cultural appropriation.

### Relationship to the casino

With the focus and connections participants made between gambling and casinos, it would be remiss to avoid discussing the relationship between Asian gambling and the role of the casino itself. As noted in the results, participants described what they felt was targeted advertising and marketing toward Asians in particular, a concern that has been noted by researchers and the media (13, 22, 60–62).

Through the interviews with participants, it was apparent that the lack of accessible social and recreational activities for Asians created a void that participants felt casinos were able to capitalize on. Participants referred to the service they receive from the casinos, in particular the language access. The inclusion of employees who speak Asian languages makes Asians feel comfortable and creates an environment they struggle to find elsewhere. The concerts were a common draw for participants who expressed desire to attend shows that showcased artists they were familiar with. By catering to the desires of Asian clientele, the casinos are able to lure in Asian immigrants seeking a place of belonging. The lives of working-class Asian immigrants described in this study are hard, stressful, and lonely. These individuals desire outlets for stress, a place where they feel comfortable and able to unwind, an escape from the reality of their daily lives. With a lack of culturally appropriate outlets, participants describe community members turning to casinos and gambling to fill this much needed gap in services. The relationship between Asian communities and local casinos is further complicated by casinos hiring bilingual Asian workers from the communities themselves.

The significance of casino buses ferrying Asians to casinos is not a finding unique to the findings of this study. Discussions of the casino buses have been occurring in other Asian ethnic communities and have even been subject to photo essays and media attention (13, 60, 63). While some may argue that casinos are offering cultural activities for these ethnic communities, the concerns of participants around the role of casinos in playing a role in encouraging Asians to gamble makes one question whether casinos are taking advantage of a vulnerable community. Blame for these marketing strategies does not rest solely on the casinos, but rather connects to the myth of the Asian gambler. Additionally, the myth of the Asian gambling culture, like most structural inequalities, is self-perpetuating. The marketing teams of casinos are influenced by the stereotype, and they target Asian populations (64). The power inequality between casinos and vulnerable Asian populations leads to more Asian gamblers thereby perpetuating the myth.

### Structural racism

Structural racism can be characterized by systems that are by design or negligence cyclical and self-propagating (65). In the United States, the vulnerability of Asian immigrants to problem gambling showcases several cyclic structures that work against low-income Asian immigrants in relation to gambling behaviors.

The distribution methods of large gambling studies in the US, often available in English only, results in an invisibility of Asian data. Beyond language access, methodology for larger studies, especially when looking at multiple race categories, fails to use appropriate means to contact and reach certain Asian demographics. Studies in English and distributed through common mainstream channels may not reach Asian immigrant communities with limited English proficiency who typically do not access these channels of communication. The lack of nuanced Asian data leads to gambling treatment and support systems that are not culturally appropriate for Asian gamblers. Failing to capture the at-risk Asian demographic leads to prevention strategies targeted at mainstream white American gamblers that overlook important root causes for Asian gamblers identified in these interviews (integration stress from social isolation and struggle to make a living). As such, these gambling interventions miss opportunities to help struggling Asian immigrants feel a sense of belonging in American society.

The lack of useful Asian data further leaves a void that has allowed the normalization of the stereotype of the Asian gambler. Even within the Asian community this myth is propagated and normalized within society. The more it is normalized, the more it can be used as an excuse by Asian gamblers themselves and by external bodies (i.e., casinos, policy makers, and researchers). The perpetuation of this stereotype both within Asian communities and externally results in a failure to examine systemic issues which may be driving Asian immigrants to gamble.

The interviews speak to the “life cycle of being an immigrant” and the possible influence this life has upon gambling behavior. As Asian immigrants struggle to make a living and experience a sense of loneliness, boredom, and isolation due to linguistic and cultural barriers, they turn to gambling for to relieve stress and find a community. Gambling as a primary recreational outlet, however, will over time lead to financial losses which can worsen financial stress and decrease a sense of belonging and acceptance in American society. Some of the gamblers may develop gambling related harms, which impacts the communities they come from, completing the cycle as the struggle intensifies.

Casinos play a significant role in the cycle of spreading Asian gambling culture. The inadequately supported social support systems within the community mean that casinos are perfectly poised to conveniently provide a place of belonging. Thereby, the casinos remain packed with Asians, creating an overly representative image that reinforces the myth of the Asian gambler. By relying on the Asian gambler image, casinos create a sense of cultural normalcy around casino gambling, despite gambling being illegal in some of the Asian countries that interview participants came from. The casinos are able to target susceptible, low-income Asians through marketing, busing, and overall environment, thereby drawing more Asians into the casino and contributing to the perpetuation of the gambling stereotype.

Without structural intervention that targets root causes discussed in the interviews, these cycles cannot be easily broken. Asians have long been recognized as an at-risk community for problem gambling, particularly those who are low-income and limited English-speaking. Yet as a society, we have failed to properly address this issue (66). Therefore, we believe that there are opportunities for change and have developed recommendations for future programs and services in the Greater Boston area.

### Opportunities for change and recommendations

Research findings have led Asian CARES to make a series of actionable recommendations, including funding ethnic-based community organizations to develop and deliver culturally and linguistically responsive problem gambling and mental health services and investing in immigrant neighborhoods by creating safe and welcoming spaces of belonging for the Asian immigrant communities where they can pursue recreational and social opportunities. While participants illuminated a painful and challenging issue within the community, they also provided wisdom and insight. The findings highlight social isolation, loneliness, and dislocation which have resulted from immigration and communities of people struggling to find and maintain employment while facing racism and discrimination.

A major recommendation by Asian CARES is a push for investing in the neighborhoods where Asian immigrants live, work, and play. By investing in these neighborhoods, vulnerable Asian communities will be able to create spaces of belonging where they can go for recreational and social opportunities.

Culturally and linguistically appropriate services provided by the trusted ethnic-based CBO like Asian CARES partners who serve as cultural brokers for their communities should be leveraged to provide services that address, alleviate, and heal this problem in the community. Participants expressed a desire to receive help and services from organizations in their community that they trusted, people who understood their culture and their lived experiences. The role of ethnic CBOs is vital in providing services tailored to the actual needs and situations of the community and for ensuring thriving communities for working-class Asian immigrant families (67–70). In terms of problem gambling, there is a limited number of existing resources for the Asian community, and while government agencies are working to be more responsive to the community, there is still a need for more services to this population. As ethnic-based CBOs serve as a safety net for vulnerable Asian communities, their knowledge and lived experience provide the expertise necessary for helping and reaching this population.

Beyond the actionable recommendations for addressing the research findings, there are also areas for future research. As noted in the limitations of our research, more focus could be placed on gathering information from other Asian ethnic groups to further illuminate the issue and highlight the nuances between ethnic groups, particularly Southeast Asian groups, who were not captured in the research. Further research into the issues of language access and marketing practices of the casinos is warranted. As casinos have in-language and culturally relevant recreational services, future studies into cultural isolation and casino marketing would be beneficial to better understand their intersection. Additionally, this research captures a picture of problem gambling from Asians living primarily in the Greater Boston area. Future research could be expanded to other ethnic communities and to other geographic regions.

Additionally, some of the recommendations of the research which were presented in the Asian CARES report (71) published by the Massachusetts Gambling Commission are being considered. In the future, as these recommendations are being implemented, a follow-up studies should examine the effects of these recommendations on the prevalence and impacts of problem gambling on Asian communities and potential changes in participant responses. They could demonstrate how CBPR research focused on addressing racial inequities and advocating for change can lead to positive outcomes for a vulnerable community.

### Limitations

Limitations of this study should be taken into consideration when reviewing the results and considering future research. Broadly, there are some limitations to qualitative research, especially considering the self-reported data. All responses from participants were based on their past recollections and could include recall bias. Additionally, a majority of the interviewees were female which may have resulted in a bias toward their perspectives rather than male perspectives. International studies have shown that in some Asian cultures, perception of gambling and acceptability of gambling behaviors can vary based on gender (18, 72). As the data was gathered during a pandemic, some of the social interactions they were referring to were currently unavailable.

The research also specifically focused on Asians in the Greater Boston region, and the potential for regional specificity should be considered before generalizing to other communities. The invisibility of Asians and Asian immigrants in gambling research is much less of an issue outside of the United States, where more focus has been placed on the habits and psychology of Asian gamblers (16, 73–75). Asian immigrant experiences in other countries may vary from the experience of Asians in the US.

Disaggregation of data is an important and often overlooked aspect of Asian research. The common practice of aggregating data often results in lumping many Asian ethnic groups into one large “Asian” umbrella. While the aim of our research was to provide insight into multiple Asian ethnic groups, time and capacity restraints on the community fieldworkers influenced the ability to gather robust data on certain ethnic groups. Due to the COVID-19 pandemic and growing racial unrest from the Atlanta shootings, community fieldworkers were stretched thin serving an increase in clients and constituents. Additionally, the demands on the ethnic communities themselves constrained the interviewing process. Therefore, sample sizes for some ethnic groups, particularly Korean and Vietnamese participants, resulted in insufficient representation for disaggregation of data. Despite this, we still found notable heterogeneity by ethnic communities that should be investigated in future research.

## Data availability statement

The original contributions presented in the study are included in the article/Supplementary material, further inquiries can be directed to the corresponding author/s.

## Ethics statement

The studies involving human participants were reviewed and approved by Tufts University Social Behavioral and Educational Research Institutional Review Board (SBER IRB). The Ethics Committee waived the requirement of written informed consent for participation.

## Author contributions

MHC was primarily responsible for the preparation and writing of this document, involved in all stages of the research including study design, data collection and analysis, and dissemination of research. BH was a co-principal investigator for the Asian CARES project, was involved in conceptualization of the project, community outreach, dissemination of the research, and contributed to the review and editing of the manuscript. DS and LL are community partners who were involved in the conceptualization, community outreach, and dissemination of the Asian CARES research, provided management of the community fieldworkers, and contributed to the review and editing of the manuscript. MY was a co-principal investigator for the Asian CARES project, was involved in conceptualization of the project, community outreach, dissemination of the research, provided management of the community fieldworkers, and contributed to the review and editing of the manuscript. MTC contributed to the review and editing of the manuscript and supervised the writing process. HR was a co-principal investigator for the Asian CARES project, responsible for all stages of the research including study design, methodology, data collection and analysis, dissemination of research, and contributed to the review and editing of the manuscript. All authors contributed to the article and approved the submitted version.

## Funding

This research was made possible by a grant (BD-19-1068-1700-134614) from the Massachusetts Gaming Commission. This project was supported by the National Center for Advancing Translational Sciences, National Institutes of Health, Award Number UL1TR002544.

## Conflict of interest

Author LL was employed by Vietnamese American Initiative for Development Inc. Authors BH, LL, DS, MY, and MTC were employed by ADAPT Coalition. Authors BH and MY were employed by Boston Chinatown Neighborhood Center (BCNC). The remaining authors declare that the research was conducted in the absence of any commercial or financial relationships that could be construed as a potential conflict of interest.

## Publisher's note

All claims expressed in this article are solely those of the authors and do not necessarily represent those of their affiliated organizations, or those of the publisher, the editors and the reviewers. Any product that may be evaluated in this article, or claim that may be made by its manufacturer, is not guaranteed or endorsed by the publisher.

## Author disclaimer

The content is solely the responsibility of the authors and does not necessarily represent the official views of the NIH.

## Footnotes

^1^Henceforth, the term “Asian” refers to Asians living in the United States unless specified otherwise. Asian(s) is used rather than Asian American(s) as many of the participants in our study are first generation immigrants who do not consider themselves as Asian American. Rather, these individuals view themselves as Asians who are in the US.
