# Supplementary material for: Unpacking the root causes of gambling in the Asian community: Contesting the myth of the Asian gambling culture
Source: Front Public Health. 2022 Nov 3;10:956956. doi: 10.3389/fpubh.2022.956956 (PMC9670317; doi:10.3389/fpubh.2022.956956)
Supplement: Supplementary file 1 [file Data_Sheet_1.PDF]

Interview Questions for Community Members

Hello. I appreciate you taking the time to meet with me today. My name is ______________ and I would like to speak with you about your thoughts on gambling in your community.

[Go over consent script]

I would like to video/audio record this interview. Do I have your permission to do this?

I would also like to take notes during the interview. Do I have your permission to do that?

Warm-up Questions:

1. How long have you lived in [insert community]?

Gambling Perception Questions:
The next questions will be about your opinions on gambling.

2. Do you think gambling is a problem in the community? Why/why not?

3. What kind of gambling activities do you see occurring in your community?
    a. What do you think are the top 3 most prevalent ways people gamble in your community? (**Probes:** lottery, casinos, sports betting, private wagering, horse racing, raffle, bingo, cultural games like mahjong, Go-Stop, or Kla Kloak, etc.)
    b. In your opinion, what are the three most prevalent ways gambling happens? (Probes: casinos, homes, underground gambling dens, on-line, lottery, race track)
    c. Do you see people playing games recreationally or is there usually more money involved?

4. What do you think are the reasons that cause people to gamble? (**Suggestions:** boredom, social isolation, lack of recreational activities, poverty, holidays/events, stress, depression, etc.)

5. Based on what you have seen, what kind of impacts has gambling had on families?
    Prompting questions if there has been an impact on the family: (Please reiterate to interviewee to not name people and to not provide identifying details of persons.)
    a. How does gambling impact stress in families?
    b. How does gambling impact violence or abuse?
    c. How does gambling impact children?
    d. How does gambling impact a person or family’s financial situation?

6. Without naming names, have you heard of situations where someone has spent so much while gambling that it has affected their financial situation? We are interested in your general opinions rather than discussing specific individuals.

   If the interviewee answers yes, ask:

   a. How did the loss affect them? (**Probes:** loss of job, difficulty paying bills, homelessness, domestic violence, shaming by family, community shunning, prostitution, trafficking, etc)
   b. What financial help did they seek due to their gambling?
      - ☐ Loans
      - ☐ Asking friends for money
      - ☐ Loan sharks (someone who offers money outside of formal the banking system)
      - ☐ Taking extra jobs
      - ☐ Pawning items
      - ☐ Other ____________
   c. What do you think could lead someone to become a chronic gambler?

7. Are you aware of the free shuttles that take people to casinos?

   If the interviewee answers yes, ask:

   In your opinion, what encourages people to take these buses to the casino? (**Probes:** it was free, to see a show, dinner, discounts, vouchers for gambling)
   In your opinion, who takes these shuttles?
   In your opinion, do you think people would still go to the casinos if there were no free shuttles?

8. Do you think that gambling behavior in your community has changed since the pandemic? If so how?

9. Do you find that it is hard to talk about problem gambling in the community?

   If the interviewee answers yes, ask:

   a. What makes it so hard to talk about?
   b. How would you combat these challenges to feel more comfortable talking about gambling?

Treatment/Solution Questions:

The next set of questions is about your opinions on treatment options and services in the community.

10. Who do you think is the first person to seek help when there are gambling problems in the home? (**Probes:** the person with gambling problems, spouse, relative, child, parent, friend, etc.)

a. Why would you choose to seek out this person? (Probes: people who can speak their language, someone who understands their culture, people they trust, etc)

11. Where would they feel comfortable seeking help? **(Probes: c**ommunity center, school, church or faith organization, social support organization, etc.)

12. How will they seek help? (Probes: Help-line, in-person one-on-one meetings, self-help groups, family counseling session, hospital, etc.)

    a. Do you think problem gamblers will call a help-line? Why/why not?

13. Currently, to your knowledge, where would people struggling with gambling go seek services and treatment?

    Prompting questions:

    a. What help options do you know of to treat gambling addiction?
    b. What resources are there in the community to help people with gambling problems?

14. In your opinions, what programs and services would best help those struggling with gambling issues?

    Prompting questions:

    a. What would help them with gambling addiction?
    b. What help would their families need? Their children?
    c. What kind of financial help would they need?
    d. What other kind of help would they need?

15. Who do you think are the trusted sources for information and resources within the community?

    Follow-up questions:

    a. What type of information do you trust?
        - ☐ Statistics
        - ☐ Personal stories
        - ☐ A mixture of both
        - ☐ Information given by professionals
        - ☐ Other ____________

    b. Where do you get your news and information from?
        - ☐ Papers
        - ☐ Ethnic news sources
        - ☐ Social media
        - ☐ Radio
        - ☐ Online forums
        - ☐ WeChat

☐ Other ______________________

16. If more resources could come into the community to help people with gambling problems, where should those resources be directed?

Organization Questions: (if the interviewee was not recruited through your organization or does not use your organization's services, skip questions 18 and 19)
The final questions have to do with your community and your experiences with [insert name of organization they use – ie BCNC, ATASK, CMAA, or VietAID].

17. When did you start coming to [insert organization] and why did you start coming here?
    (Ex – When did you start coming to VietAID and why did you start coming here?)

18. How has the organization helped you in the past?
    a. What kinds of activities or programs have you participated in?
    b. How has your family used this organization?
    c. Are there specific kinds of one-on-one help you have gotten here?
    d. Problems they have helped you solve? How?
    e. What kinds of programs or services would help gambling issues in your particular community?

Demographic questions:

I'd now like to ask you a few questions about yourself.

19. What race and ethnicity do you identify with?
    - ☐ Chinese
    - ☐ Taiwanese
    - ☐ Korean
    - ☐ Vietnamese
    - ☐ Cambodian
    - ☐ Indian
    - ☐ Bengali
    - ☐ Nepali
    - ☐ Thai
    - ☐ Other _____________

20. What gender do you identify with?
    - ☐ Male
    - ☐ Female
    - ☐ Transgender
    - ☐ Non-binary
    - ☐ Prefer not to specify

☐ Other ___________

21. What is your highest level of education?

☐ No formal education
☐ Grade School
☐ High School or equivalent
☐ Vocational or trade school
☐ Associates degree
☐ Bachelor’s degree
☐ Master’s degree or professional degree
☐ Doctorate degree

22. What do you consider your primary language?

☐ Mandarin
☐ Cantonese
☐ Hindi
☐ Bengala
☐ Nepali
☐ Korean
☐ Vietnamese
☐ Khmer
☐ English
☐ Other _________

23. What is your level of English language proficiency?

☐ Fluent
☐ High
☐ Medium
☐ Limited
☐ None

24. What industry do you work in?

25. What is your age? ___________ years old (based on US age system)

26. Were you born in the US, or did you immigrate here? If you immigrated, how many years have you lived in the US? <u>Please *do not* tell us your immigration status</u>. We are interested in how long you have been in the US not your legal status.

______________years

27. How many people do you live with?

28. How many children do you have?

Thank you for your time.
